# Supplementary material for: The Gut Bacteria Dysbiosis Contributes to Chronic Graft-Versus-Host Disease Associated With a Treg/Th1 Ratio Imbalance
Source: Front Microbiol. 2022 Sep 8;13:813576. doi: 10.3389/fmicb.2022.813576 (PMC9493085; doi:10.3389/fmicb.2022.813576)
Supplement: Supplementary file 1 [file Data_Sheet_1.zip › P101SC18090073-01-B1-3-4_result/05.WebShow/taxa_charts_html/charts/47fzWMQZDoyUUdxiOzRYqE6SEi54z4_legend.pdf]

k__Bacteria;p__Bacteroidetes;c__Bacteroidia;o__Bacteroidales;f__Bacteroidaceae;g__Bacteroides
k__Bacteria;p__Bacteroidetes;c__Bacteroidia;o__Bacteroidales;f__Muribaculaceae;g__
k__Bacteria;p__Bacteroidetes;c__Bacteroidia;o__Bacteroidales;f__Muribaculaceae;g__Muribaculum
k__Bacteria;p__Bacteroidetes;c__Bacteroidia;o__Bacteroidales;f__Prevotellaceae;g__Alloprevotella
k__Bacteria;p__Bacteroidetes;c__Bacteroidia;o__Bacteroidales;f__Rikenellaceae;g__Alistipes
k__Bacteria;p__Firmicutes;c__Clostridia;o__Clostridiales;f__Lachnospiraceae;Other
k__Bacteria;p__Firmicutes;c__Clostridia;o__Clostridiales;f__Lachnospiraceae;g__
k__Bacteria;p__Firmicutes;c__Clostridia;o__Clostridiales;f__Lachnospiraceae;g__Blautia
k__Bacteria;p__Firmicutes;c__Clostridia;o__Clostridiales;f__Lachnospiraceae;g__Lachnoclostridium
k__Bacteria;p__Firmicutes;c__Clostridia;o__Clostridiales;f__Lachnospiraceae;g__Roseburia
k__Bacteria;p__Firmicutes;c__Clostridia;o__Clostridiales;f__Lachnospiraceae;g__unidentified_Lachnospiraceae
k__Bacteria;p__Firmicutes;c__Clostridia;o__Clostridiales;f__Ruminococcaceae;Other
k__Bacteria;p__Firmicutes;c__Clostridia;o__Clostridiales;f__Ruminococcaceae;g__Oscillibacter
k__Bacteria;p__Firmicutes;c__Clostridia;o__Clostridiales;f__Ruminococcaceae;g__Ruminiclostridium
k__Bacteria;p__Firmicutes;c__Clostridia;o__Clostridiales;f__Ruminococcaceae;g__unidentified_Ruminococcaceae
k__Bacteria;p__Proteobacteria;c__Deltaproteobacteria;o__Desulfovibrionales;f__Desulfovibrionaceae;g__Desulfovibrio
k__Bacteria;p__Proteobacteria;c__Gammaproteobacteria;o__Enterobacteriales;f__Enterobacteriaceae;Other
k__Bacteria;p__Proteobacteria;c__Gammaproteobacteria;o__Enterobacteriales;f__Enterobacteriaceae;g__Proteus
k__Bacteria;p__Proteobacteria;c__Gammaproteobacteria;o__Enterobacteriales;f__Enterobacteriaceae;g__unidentified_Enterobacteriaceae
